# Risk Communication and Ebola-Specific Knowledge and Behavior during 2014–2015 Outbreak, Sierra Leone

**DOI:** 10.3201/eid2402.171028

**Published:** 2018-02

**Authors:** Maike Winters, Mohamed F. Jalloh, Paul Sengeh, Mohammad B. Jalloh, Lansana Conteh, Rebecca Bunnell, Wenshu Li, Zangin Zeebari, Helena Nordenstedt

**Affiliations:** Karolinska Institutet, Stockholm, Sweden (M. Winters, M.F. Jalloh, Z. Zeebari, H. Nordenstedt);; Centers for Disease Control and Prevention, Atlanta, Georgia, USA (M.F. Jalloh, R. Bunnell, W. Li);; FOCUS1000, Freetown, Sierra Leone (P. Sengeh, M.B. Jalloh);; Ministry of Health and Sanitation Sierra Leone, Freetown (L. Conteh)

**Keywords:** Risk communication, global health security, Ebola, Sierra Leone

## Abstract

We assessed the effect of information sources on Ebola-specific knowledge and behavior during the 2014–2015 Ebola virus disease outbreak in Sierra Leone. We pooled data from 4 population-based knowledge, attitude, and practice surveys (August, October, and December 2014 and July 2015), with a total of 10,604 respondents. We created composite variables for exposures (information sources: electronic, print, new media, government, community) and outcomes (knowledge and misconceptions, protective and risk behavior) and tested associations by using logistic regression within multilevel modeling. Exposure to information sources was associated with higher knowledge and protective behaviors. However, apart from print media, exposure to information sources was also linked to misconceptions and risk behavior, but with weaker associations observed. Knowledge and protective behavior were associated with the outbreak level, most strongly after the peak, whereas risk behavior was seen at all levels of the outbreak. In future outbreaks, close attention should be paid to dissemination of information.

West Africa detected its first case of Ebola virus disease (EVD) in March 2014 within the forest region of Guinea; shortly thereafter, Liberia and Sierra Leone detected cases of the disease (*1*)*.* More than 28,600 confirmed, probable, and suspected cases of EVD in Guinea, Sierra Leone, and Liberia led to >11,300 deaths by the time the World Health Organization declared the epidemic over (*1*).

Global health security relies on all countries having the capacity to rapidly detect and control public health threats at their source. Accurate and timely risk communication efforts are essential for effective disease control efforts, and therefore for global health security. A key aspect of the Ebola response was to engage and educate the public on preventing transmission and seeking early medical care (*2*). In Sierra Leone, the Ministry of Health and Sanitation established a social mobilization pillar in June 2014 for the development and coordination of community engagement strategies to contain the spread of EVD (*3*)*.*

The EVD epidemic dominated headlines around the world (*4*). Mass and social media are believed to have played a role in disseminating incorrect information, which could have influenced EVD knowledge and health-seeking behavior (*5*)*.* In Sierra Leone, 88% of persons surveyed reportedly received information about EVD through the radio, which was the preferred means of receiving EVD information (*6*). The influence of media on disease or health outcomes has previously been studied in different contexts (*7**–**9*). In Burkina Faso, a large trial examined the influence of radio campaigns on child mortality (*10*). Midline results from the trial reported moderate improvement in seeking care for diarrhea-related diseases (*11*).

Apart from media, information sources such as educational campaigns and community leaders are thought to be essential during an infectious disease outbreak (*12*). In Sierra Leone, 41% of survey respondents received information about EVD through religious leaders in the initial phase of the EVD outbreak (*6*).

Mathematical models have shown that accurate reporting may slow down the initial phase of an infectious disease outbreak and could lower the number of cases during the peak of an outbreak, but inaccurate reporting could lead to an increase in the number of cases (*13*). Furthermore, awareness and knowledge during an epidemic are attributable to a wide availability of information, highlighting “the crucial role of mass media and educational campaigns” (*14*)*.* However, mathematical models are limited in that they represent an ideal, standardized situation and will never be better than the variables included in the model. Knowledge, attitude, and practice (KAP) surveys are an established way of empirically measuring persons’ knowledge and self-reported behavior in relation to a disease (*15*). A KAP survey administered in Liberia, for instance, found that even though awareness about EVD was high, knowledge was poor (*16*). In Guinea, 96% of respondents had heard of EVD, but only 36% had comprehensive knowledge (accepting 3 methods of prevention and rejecting 3 common misconceptions) about the disease (*17*). A study in Nigeria revealed that only half of the respondents knew that EVD was caused by a virus (*18*). All studies recognize that media and other information sources could play a crucial role in enhancing EVD knowledge (*16**–**18*).

In Sierra Leone, the country with the most EVD cases, the roles that different types of information sources played in influencing knowledge and behavior during the EVD outbreak remain unknown. If, and to what extent, knowledge plays a mediating role in changing behavior, especially in an infectious disease outbreak, is debated (*19**,**20*). Furthermore, the level of outbreak, or the intensity of an outbreak, is thought to have an effect on knowledge and behavior (*21*), but this factor has not yet been studied in Sierra Leone. Therefore, we assessed the effects of different information sources on Ebola-specific knowledge and behavior and how the level of the outbreak affected these factors.

## Methods

We administered 4 KAP surveys from August 2014 through July 2015 in Sierra Leone (Technical Appendix Figure 1). In all KAP surveys, we applied multistage cluster sampling. KAP 1 was conducted in 9 districts from all 4 regions of Sierra Leone. KAPs 2–4 (October 2014, December 2014, and July 2015) covered all 14 districts in the country. The 2004 Sierra Leone Population and Housing Census List of Enumeration Areas (http://www.sierra-leone.org/Census/ssl_final_results.pdf) served as the sampling frame for random selection of enumeration areas from the districts. Within enumeration areas, we used the random walk method, a form of systematic random sampling, to select households. In each selected household, we conducted 2 interviews: first with the head of the household, and second with either a woman or a young person 15–24 years of age who was randomly selected. All 4 KAP surveys were designed to produce national- and regional- level estimates at a 95% confidence level within a 2.5% margin of error for national estimates and a 3.5% margin of error for regional estimates (Technical Appendix Table 1).

We captured exposure to a source of information in the KAP surveys with the question: “Through what means/ways did you learn about Ebola?” Categories were not mutually exclusive; data collectors ticked “yes” in all boxes that applied. Furthermore, more response options were included in later versions of the KAP surveys. Five categories reflected the different information sources: electronic media (radio and television); print media (newspapers, brochures, and other print materials); new media (mobile phones, text messages, and internet); government (house visits by health workers); and community (religious and traditional leaders, megaphone public announcements, community meetings, friends, and relatives). We categorized number of exposures into 0–1 source, 2, 3, and 4–5 sources. The sample of respondents exposed to 0 or 5 sources was low (n = 38 for 0 sources and n = 265 for 5 sources). Therefore, we combined 0 with 1 and 5 with 4. The category “level of outbreak” was broken down into before the peak, peak, after peak, and no transmission for each region. We consulted World Health Organization data to create this variable for every region in all 4 KAP surveys (*1*) (Technical Appendix Table 2).

We tested Ebola-specific knowledge as both an outcome of exposure to information and a mediating factor in the association between information exposure and behavior (Technical Appendix Figure 2). We assessed Ebola-specific knowledge in the KAP surveys with 2 open-ended and 5 closed-ended questions, resulting in 2 scores, 1 for knowledge and 1 for misconception (Technical Appendix Table 3). In the knowledge score, a maximum score of 8 points was possible in KAP 1–4. In the misconception score, 12 points was the maximum in KAP 1 and 13 points in KAP 2–4, due to an additional response option. We dichotomized the scores based on the means (*22*).

We measured Ebola-specific behavior by 2 open-ended questions and created 2 scores, 1 for protective behavior and 1 for risk behavior. For the protective behavior score, respondents could tally a maximum of 7 in KAP 1; in KAP 2–4, the maximum was 9. The risk behavior score had a maximum of 9 points in KAP 1 and 11 in KAP 2–4. We again dichotomized the scores based on the means.

Working with information from previous KAP survey analyses (*17**,**18*), we chose the following covariates as potential confounders: gender (male, female); age (13–20, 21–35, 36–49, >50 years); education (no education, primary education, and secondary and above); religion (Islam, Christianity); and region (Northern Province, Eastern Province, Southern Province, Western Area).

### Statistical Analyses

To account for the high correlation between cases over time and region, we applied multilevel modeling (*23*). We pooled all data from the 4 KAP surveys and created clusters that grouped the specific KAP survey and the district. In KAP 1, there were 9 districts, and in KAP 2–4 there were 14 districts, for a total of 51 clusters. We collected samples proportionally to the district size in the population, and the response rate was 98%. Therefore, the estimators were considered unbiased, and we did not apply the original survey weights. We envisioned the same approach for creating clusters for the analyses regarding the intensity of the outbreak, but because of the high correlation of the level of outbreak variable with the time point of the KAP survey (r = 0.82), the cluster level included only districts. We estimated associations with odds ratios (ORs) and their corresponding 95% CIs. To test whether knowledge played a mediating role on the outcome, we performed mediation analyses using the mediated effect model (*24*). We obtained the β coefficients for A and B (Technical Appendix Figure 2) from the fully adjusted models tested in the multilevel modeling, multiplied them, and calculated SEs. Statistical significance of the mediated effect was determined with the χ^2^ distribution within 1 df. We used SPSS version 22 (Armonk, NY, USA) for the analyses and set α to 0.05 for statistical significance.

The Sierra Leone Research and Scientific Review Committee granted ethicals permission for all KAP surveys. The KAP 2–4 assessments were further reviewed and approved by the US Centers for Disease Control and Prevention.

## Results

The overall response rate of the 4 KAP surveys was 98%. The pooled sample of KAP 1–4 consisted of 10,604 respondents. Because of missing values for some variables, we excluded 95 respondents (0.9%), making the total study sample 10,509. The age distribution was similar in the different regions, with those 21–35 years of age representing the largest group (Table 1). The total sample had an even distribution of men and women (49.1% men, 50.9% women). In all regions apart from the Northern Province, most of the sample had at least primary schooling. Two thirds of the respondents were affiliated with Islam (67.3%). EVD messages were received mostly through electronic media (94.1%) and community sources (59.5%) (Table 2); a substantially smaller portion were exposed to print (8.7%) and new media (15.5%). Government information campaigns reached 47.7% of respondents. Most respondents were exposed to >2 different types of information sources (72.6%).

**Table 1:** Demographics of respondents to Ebola knowledge, attitude, and practice surveys, by region, Sierra Leone, 2014–2015

Category	No. (%) respondents				
	Northern Province	Eastern Province	Southern Province	Western Area	Total
Age, y					
15–20	854 (21.2)	505 (24.4)	440 (23.4)	539 (21.3)	2,338 (22.2)
21–35	1,392 (34.6)	706 (34.1)	657 (35.0)	930 (36.7)	3,685 (35.1)
36–49	909 (22.6)	476 (23.0)	416 (22.2)	629 (24.8)	2,430 (23.1)
>50	869 (21.6)	384 (18.5)	365 (19.4)	438 (17.2)	2,056 (19.6)
Sex					
M	2,112 (52.5)	972 (46.9)	898 (47.8)	1,182 (46.6)	5,163 (49.1)
F	1,912 (47.5)	1,100 (53.1)	980 (52.2)	1,354 (53.4)	5,346 (50.9)
Education					
No education	1,755 (43.6)	758 (36.6)	496 (26.4)	508 (20.0)	3,517 (33.5)
Primary	733 (18.2)	488 (23.6)	367 (19.5)	371 (14.7)	1,959 (18.6)
Secondary and above	1,536 (38.2)	825 (39.8)	1,015 (54.1)	1,657 (65.3)	5,033 (47.9)
Religion					
Islam	3,318 (82.5)	1,320 (63.7)	1,104 (58.8)	1,335 (52.6)	7,077 (67.3)
Christianity	706 (17.5)	751 (36.3)	774 (41.2)	1,201 (47.4)	3,432 (32.7)
Total	4,024	2,071	1,878	2,536	10,509

**Table 2:** Association between information exposure and knowledge among respondents to Ebola knowledge, attitude, and practice surveys, Sierra Leone, 2014–2015*

Category	No. (%) respondents	Crude OR (95% CI)	p value†	Adjusted OR‡ (95% CI)	p value†
Source of information					
Electronic media					
Yes	9,894 (94.1)	1.84 (1.54–2.19)	<0.001	1.75 (1.46–2.09)	<0.001
No	615 (5.9)	1.0 (Reference)		1.0 (Reference)	
Print media					
Yes	918 (8.7)	1.96 (1.67–2.31)	<0.001	1.47 (1.24–1.75)	<0.001
No	9,591 (91.3)	1.0 (Reference)		1.0 (Reference)	
New media					
Yes	1,627 (15.5)	1.91 (1.68–2.17)	<0.001	1.41 (1.23–1.61)	<0.001
No	8,882 (84.5)	1.0 (Reference)		1.0 (Reference)	
Government					
Yes	5,011 (47.7)	1.77 (1.62–1.94)	<0.001	1.56 (1.42–1.71)	<0.001
No	5,498 (52.3)	1.0 (Reference)		1.0 (Reference)	
Community					
Yes	6,248 (59.5)	1.63 (1.49–1.78)	<0.001	1.44 (1.31–1.59)	<0.001
No	4,261 (40.5)	1.0 (Reference)		1.0 (Reference)	
No. exposures					
0–1 source	2,878 (27.4)	1.0 (Reference)		1.0 (Reference)	
2 sources	3,408 (32.5)	1.41 (1.26–1.56)	<0.001	1.37 (1.23–1.53)	<0.001
3 sources	3,115 (29.6)	2.18 (1.94–2.45)	<0.001	2.09 (1.86–2.35)	<0.001
4–5 sources	1,108 (10.5)	4.39 (3.65–5.28)	<0.001	3.83 (3.17–4.61)	<0.001

*OR, odds ratio. †Wald statistical p value from the multilevel model.  ‡Adjusted for region, gender, age, religion, educational level, level of outbreak and all other information exposures.

### Knowledge and Protective Behavior Scores

Exposure to any type of information sources was statistically significantly associated with increased knowledge in both the crude and adjusted models (Table 2). Electronic media showed the strongest association in the adjusted model (adjusted OR [aOR] 1.75, 95% CI 1.46–2.09). Strong associations could also be seen between all types of information sources and protective behavior in all adjusted models (Table 3). New media showed the strongest association with protective behavior (aOR 2.15, 95% CI 1.87–2.48). We identified a clear dose-response association between number of sources and knowledge (4–5 sources aOR 3.83, 95% CI 3.17–4.61) and protective behavior (4–5 sources aOR 6.77, 95% CI 5.53–8.28).

**Table 3:** Association between information exposure and protective behavior among respondents to Ebola knowledge, attitude, and practice surveys, Sierra Leone, 2014–2015*

Category	No. (%) respondents	Crude OR (95% CI)	p value†	Adjusted OR‡ (95% CI)	p value†	Adjusted OR§ (95% CI)	p value†
Source of information							
Electronic media							
Yes	9,894 (94.1)	2.17 (1.80–2.63)	<0.001	2.14 (1.76–2.59)	<0.001	2.00 (1.65–2.43)	<0.001
No	615 (5.9)	1.0 (Reference)		1.0 (Reference)		1.0 (Reference)	
Print media							
Yes	918 (8.7)	2.43 (2.07–2.87)	<0.001	1.71 (1.44–2.04)	<0.001	1.65 (1.38–1.97)	<0.001
No	9,591 (91.3)	1.0 (Reference)		1.0 (Reference)		1.0 (Reference)	
New media							
Yes	1,627 (15.5)	2.98 (2.61–3.40)	<0.001	2.20 (1.91–2.53)	<0.001	2.15 (1.87–2.48)	<0.001
No	8,882 (84.5)	1.0 (Reference)		1.0 (Reference)		1.0 (Reference)	
Government							
Yes	5,011 (47.7)	2.15 (1.96–2.35)	<0.001	1.79 (1.62–1.96)	<0.001	1.70 (1.55–1.87)	<0.001
No	5,498 (52.3)	1.0 (Reference)		1.0 (Reference)		1.0 (Reference)	
Community							
Yes	6,248 (59.5)	2.03 (1.85–2.22)	<0.001	1.72 (1.56–1.90)	<0.001	1.65 (1.49–1.82)	<0.001
No	4,261 (40.5)	1.0 (Reference)		1.0 (Reference)		1.0 (Reference)	
No. exposures							
0–1 source	2,878 (27.4)	1.0 (Reference)		1.0 (Reference)		1.0 (Reference)	
2 sources	3,408 (32.5)	1.46 (1.30–1.63)	<0.001	1.43 (1.28–1.60)	<0.001	1.38 (1.23–1.54)	<0.001
3 sources	3,115 (29.6)	3.10 (2.75–3.49)	<0.001	3.02 (2.68–3.41)	<0.001	2.78 (2.46–3.14)	<0.001
4–5 sources	1,108 (10.5)	8.60(7.06–10.49)	<0.001	7.76 (6.35–9.48)	<0.001	6.77 (5.53–8.28)	<0.001

*OR, odds ratio †Wald statistical p value from the multilevel model. ‡Adjusted for region, gender, age, religion, educational level, level of outbreak, and all other information exposures.  §Adjusted for region, gender, age, religion, educational level, level of outbreak, and all other information exposures, plus knowledge and misconceptions.

### Misconceptions and Risk Behavior

All sources of information apart from print media were significantly associated with misconceptions; however, the point estimates were substantially lower than in the knowledge models (Table 4). After adjusting for confounders, information from electronic media showed the strongest association with misconceptions (aOR 1.42, 95% CI 1.18–1.70). In the models testing the association between information sources and risk behavior, results for electronic and print media were not significant (Table 5). New media, governmental campaigns, and community sources did have an association, with community sources showing the strongest association with misconceptions (aOR 1.34, 95% CI 1.22–1.47).

**Table 4:** Association between information exposure and misconceptions among respondents to Ebola knowledge, attitude, and practice surveys, Sierra Leone, 2014–2015*

Category	No. (%) respondents	Crude OR (95% CI)	p value†	Adjusted OR‡ (95% CI)	p value†
Type of information					
Electronic media					
Yes	9,894 (94.1)	1.29 (1.08–1.54)	0.005	1.42 (1.18–1.70)	<0.001
No	615 (5.9)	1.0 (Reference)		1.0 (Reference)	
Print media					
Yes	918 (8.7)	1.03 (0.88–1.21)	0.734	0.98 (0.83–1.15)	0.729
No	9,591 (91.3)	1.0 (Reference)		1.0 (Reference)	
New media					
Yes	1,627 (15.5)	1.22 (1.07–1.38)	0.002	1.17 (1.03–1.34)	0.020
No	8,882 (84.5)	1.0 (Reference)		1.0 (Reference)	
Government					
Yes	5,011 (47.7)	1.35 (1.24–1.48)	<0.001	1.26 (1.14–1.38)	<0.001
No	5,498 (52.3)	1.0 (Reference)		1.0 (Reference)	
Community					
Yes	6,248 (59.5)	1.46 (1.33–1.60)	<0.001	1.39 (1.26–1.53)	<0.001
No	4,261 (40.5)	1.0 (Reference)		1.0 (Reference)	
No. exposures					
0–1 source	2,878 (27.4)	1.0 (Reference)		1.0 (Reference)	
2 sources	3,408 (32.5)	1.04 (0.94–1.17)	0.400	1.07 (0.95–1.19)	0.262
3 sources	3,115 (29.6)	1.62 (1.44–1.83)	<0.001	1.67 (1.47–1.88)	<0.001
4–5 sources	1,108 (10.5)	1.73 (1.45–2.05)	<0.001	1.86 (1.56–2.22)	<0.001

*OR, odds ratio. †Wald statistical p value from the multilevel model. ‡Adjusted for region, gender, age, religion, educational level, level of outbreak, and all other information exposures.

**Table 5:** Association between information exposure and risk behavior among respondents to Ebola knowledge, attitude, and practice surveys, Sierra Leone, 2014–2015*

Category	No. (%) respondents	Crude OR (95% CI)	p value†	Adjusted OR‡ (95% CI)	p value†	Adjusted OR§ (95% CI)	p value†
Type of information							
Electronic media							
Yes	9,894 (94.1)	1.01 (0.84–1.20)	0.955	1.10 (0.92–1.31)	0.300	1.07 (0.89–1.27)	0.480
No	615 (5.9)	1.0 (Reference)		1.0 (Reference)		1.0 (Reference)	
Print media							
Yes	918 (8.7)	1.10 (0.95–1.28)	0.193	1.03 (0.88–1.20)	0.716	1.03 (0.89–1.20)	0.692
No	9,591 (91.3)	1.0 (Reference)		1.0 (Reference)		1.0 (Reference)	
New media							
Yes	1,627 (15.5)	1.27 (1.13–1.42)	<0.001	1.23 (1.09–1.39)	<0.001	1.22 (1.08–1.37)	0.002
No	8,882 (84.5)	1.0 (Reference)		1.0 (Reference)		1.0 (Reference)	
Government							
Yes	5,011 (47.7)	1.37 (1.26–1.50)	<0.001	1.25 (1.14–1.37)	<0.001	1.23 (1.12–1.34)	<0.001
No	5,498 (52.3)	1.0 (Reference)		1.0 (Reference)		1.0 (Reference)	
Community							
Yes	6,248 (59.5)	1.47 (1.34–1.61)	<0.001	1.37 (1.25–1.51)	<0.001	1.34 (1.22–1.47)	<0.001
No	4,261 (40.5)	1.0 (Reference)		1.0 (Reference)		1.0 (Reference)	
No. exposures							
0–1 source	2,878 (27.4)	1.0 (Reference)		1.0 (Reference)		1.0 (Reference)	
2 sources	3,408 (32.5)	1.18 (1.06–1.32)	0.003	1.19 (1.06–1.33)	0.002	1.18 (1.06–1.32)	0.003
3 sources	3,115 (29.6)	1.61 (1.43–1.80)	<0.001	1.63 (1.45–1.83)	<0.001	1.57 (1.40–1.77)	<0.001
4–5 sources	1,108 (10.5)	1.83 (1.57–2.14)	<0.001	1.95 (1.66–2.28)	<0.001	1.87 (1.59–2.18)	<0.001

*OR, odds ratio. †Wald statistical p value from the multilevel model. ‡Adjusted for region, gender, age, religion, educational level, level of outbreak, and all other information exposures. §Adjusted for all of the above, plus knowledge and misconceptions.

### Knowledge as Mediator

Knowledge played a mediating role in the association between all different information sources and protective behavior. In the analyses for risk behavior, all information sources apart from print media demonstrated mediation through misconceptions (Table 6). Electronic media had no direct link with risk behavior but still had an effect on risk behavior by influencing misconceptions.

**Table 6:** Mediation analyses for knowledge, misconceptions, protective behavior, and risk behavior among respondents to Ebola knowledge, attitude, and practice surveys, Sierra Leone, 2014–2015*

Category	Beta A (SE)	Beta B (SE)	Beta AB (SE)	p value† AB
Mediation analyses for knowledge and protective behavior				
Type of information				
Electronic media	0.558 (0.091)	0.662 (0.047)	0.369 (0.066)	<0.001
Print media	0.387 (0.088)	0.662 (0.047)	0.256 (0.061)	<0.001
New media	0.342 (0.069)	0.662 (0.047)	0.226 (0.048)	<0.001
Government	0.444 (0.048)	0.662 (0.047)	0.294 (0.038)	<0.001
Community	0.365 (0.049)	0.662 (0.047)	0.242 (0.037)	<0.001
No. sources				
0–1 source				
2 sources	0.317 (0.055)	0.664 (0.047)	0.210 (0.039)	<0.001
3 sources	0.737 (0.060)	0.664 (0.047)	0.489 (0.052)	<0.001
4–5 sources	1.341 (0.096)	0.664 (0.047)	0.890 (0.090)	<0.001
Mediation analyses for misconceptions and risk behavior				
Type of information				
Electronic media	0.350 (0.092)	0.400 (0.047)	0.140 (0.040)	<0.001
Print media	−0.022 (0.084)	0.400 (0.047)	−0.009 (0.034)	0.791
New media	0.157 (0.068)	0.400 (0.047)	0.063 (0.028)	0.024
Government	0.227 (0.049)	0.400 (0.047)	0.091 (0.022)	<0.001
Community	0.329 (0.050)	0.400 (0.047)	0.132 (0.025)	<0.001
No. sources				
0–1 source				
2 sources	0.063 (0.056)	0.400 (0.047)	0.025 (0.023)	0.277
3 sources	0.510 (0.062)	0.400 (0.047)	0.204 (0.034)	<0.001
4–5 sources	0.622 (0.089)	0.400 (0.047)	0.249 (0.046)	<0.001

*Beta coefficients of the adjusted ORs (adjusted for region, gender, age, religion, educational level, level of outbreak and all other information sources): A, B refer to the paths in Technical Appendix Figure 2 (https://wwwnc.cdc.gov/EID/article/24/2/17-1028-Techapp1.pdf); AB is the multiplication of A and B. OR, odds ratio. †χ^2^ test with 1 df.

### Level of Outbreak

Knowledge was significantly associated with all levels of the outbreak; with higher transmission, more respondents showed higher knowledge, although at no transmission of EVD, the association was weak (Table 7). Protective behavior also increased with the level of outbreak, but at the point of no transmission, the association decreased and was no longer significant (aOR 1.14, 95% CI 0.92–1.42). Misconceptions seemed to decline during the peak (aOR 0.76, 95% CI 0.64–0.91), and had no association after the peak (aOR 1.00, 95% CI 0.86–1.16). The stage of no transmission, however, was strongly associated with misconceptions (aOR 1.42, 95% CI 1.15–1.76). For risk behavior, all associations were significant, with the strongest one being at the peak of the outbreak (aOR 1.71, 95% CI 1.43–2.04), with a declining trend over time.

**Table 7:** Association between level of outbreak, knowledge, and behavior among respondents to Ebola knowledge, attitude, and practice surveys, Sierra Leone, 2014–2015*

Level of outbreak	No. (%)	Crude OR (95% CI)	p value†	Adjusted OR 1‡ (95% CI)	p value†	Adjusted OR 2§ (95% CI)	p value†
Association between level of outbreak and knowledge							
Before peak	1,095 (10.4)	1.0 (Reference)		1.0 (Reference)		1.0 (Reference)	
Peak	1,399 (13.3)	1.46 (1.23–1.72)	<0.001	1.26 (1.06–1.50)	0.008	1.31 (1.10–1.56)	0.002
After peak	6,995 (66.6)	2.06 (1.80–2.36)	<0.001	1.68 (1.46–1.93)	<0.001	1.83 (1.59–2.11)	<0.001
No transmission	1,020 (9.7)	1.73 (1.42–2.10)	<0.001	1.38 (1.13–1.69)	0.002	1.51 (1.23–1.86)	<0.001
Association between level of outbreak and protective behavior							
Before peak	1,095 (10.4)	1.0 (Reference)		1.0 (Reference)		1.0 (Reference)	
Peak	1,399 (13.3)	1.35 (1.14–1.61)	0.001	1.18 (0.98–1.41)	0.077	1.20 (0.98–1.43)	0.054
After peak	6,995 (66.6)	1.67 (1.45–1.92)	<0.001	1.38 (1.19–1.60)	<0.001	1.46 (1.26–1.69)	<0.001
No transmission	1,020 (9.7)	1.50 (1.23–1.82)	<0.001	1.08 (0.87–1.34)	0.470	1.14 (0.92–1.42)	0.229
Association between level of outbreak and misconceptions							
Before peak	1,095 (10.4)	1.0 (Reference)		1.0 (Reference)		1.0 (Reference)	
Peak	1,399 (13.3)	0.78 (0.66–0.93)	0.004	0.77 (0.65–0.92)	0.004	0.76 (0.64–0.91)	0.003
After peak	6,995 (66.6)	1.13 (0.98–1.30)	0.104	1.03 (0.89–1.20)	0.668	1.00 (0.86–1.16)	0.994
No transmission	1,020 (9.7)	1.64 (1.33–2.02)	<0.001	1.48 (1.20–1.83)	<0.001	1.42 (1.15–1.76)	0.001
Association between level of outbreak and risk behavior							
Before peak	1,095 (10.4)	1.0 (Reference)		1.0 (Reference)		1.0 (Reference)	
Peak	1,399 (13.3)	1.75 (1.47–2.08)	<0.001	1.72 (1.44–2.05)	<0.001	1.71 (1.43–2.04)	<0.001
After peak	6,995 (66.6)	1.67 (1.45–1.92)	<0.001	1.52 (1.31–1.75)	<0.001	1.48 (1.28–1.71)	<0.001
No transmission	1,020 (9.7)	1.50 (1.23–1.82)	<0.001	1.33 (1.09–1.62)	0.005	1.28 (1.05–1.56)	0.017

*OR, odds ratio. †Wald statistical p value from the multilevel model. ‡Adjusted for all information exposures. §Adjusted for all information exposures, gender, region, age, religion, and educational level.

## Discussion

The results of this study clearly show that exposure to different types of information sources was associated with increased knowledge and protective behaviors during the EVD epidemic in Sierra Leone. A strong dose-response association was seen between information exposure and knowledge and protective behavior. However, exposure to all information sources (apart from electronic and print media) was also significantly associated with misconceptions and risk behavior. For all sources, apart from print media, the association between information exposure and behavior was mediated by knowledge. Knowledge had a significant association with level of outbreak at all stages of the outbreak. Protective behavior was significantly associated with level of outbreak at the peak and after the peak but no longer showed an association at the point of no transmission. Misconceptions showed a decline at first but increased toward the end of the outbreak. Risk behavior was prevalent during the entire course of the outbreak, but with a declining trend.

The results reinforce the importance of radio in Sierra Leone; electronic media (which consists mainly of radio) had the strongest association with knowledge and protective behavior but also with misconceptions. Even though electronic media were not directly linked with risk behavior, there was a mediating effect on risk behavior through misconceptions. In 2013, Sierra Leone had a literacy rate of only 46% (*25*), which makes radio the preferred and most accessible source of information for a large part of the population (78% have access to radio) (*26*). Other studies have also reported the importance of radio in promoting knowledge in low-income settings (*11**,**27**,**28*). Nonetheless, the influence of radio on promoting protective behavior in these studies was not as strong (*11*) or did not have an association at all (*28*). The setting of our study, an infectious disease outbreak with high death rates, might have provided more urgency and a different risk perception and behavior change pathway than other studies (*20*). Furthermore, as part of the EVD response, radio was used as a channel to disseminate messages from community and religious leaders (*3*). An additive effect of different information sources (such as community and government) coming together in radio could explain the strong associations.

The finding that both knowledge and misconceptions and both protective and risk behavior were linked to most of the information sources could be explained by the presence of contradicting messages during the outbreak. Previous studies have reported a high prevalence of misconceptions, which have led to rejection of interventions during the EVD epidemic (*12**,**29**,**30*). Furthermore, correct and incorrect explanations of a disease might coexist in a community. In Guinea, for instance, 83% of respondents believed that a virus caused EVD, but at the same time, 36% believed that higher powers were involved in causing the outbreak (*17*). This finding could explain, in part, some of the results in our study, in which community sources were most strongly associated with risk behavior. Community sources were employed mostly in areas with high EVD transmission. Selection bias could, therefore, be an explanation of the observed associations. Furthermore, the temporal relationship between community sources and the reported risk behaviors cannot be ascertained.

Print media showed a different pattern from other information sources in the models. Exposure to print media was associated with both knowledge and protective behavior but not with misconceptions and risk behavior. A reason for this finding might be that those who read newspapers and brochures differ from the general population of Sierra Leone in that they are literate and have the means to buy a newspaper. Only 11% of the population reads newspapers on a weekly basis (*26*). Educational level was taken into account in the analyses, but residual confounding cannot be excluded.

We found a clear dose-response association for knowledge and protective behavior. Other studies highlight this finding as well; exposure to multiple information sources increases the chances of demonstrating higher knowledge and protective behavior (*31**–**34*).

Previous studies show conflicting results as to whether knowledge is needed for behavioral change (*19*). From the results of our study, it seems that behavioral change can be achieved through directly influencing behavior as well as through a mediation effect of improvement in knowledge. Even though having knowledge appears to be beneficial for adopting protective behaviors, misconceptions mediate the effect of risk behavior. This connection was also seen in reality during the EVD outbreak in West Africa, where misconceptions about the virus led to sometimes violent resistance to public health measures (*35*).

In the analysis of the association between level of outbreak and knowledge and behavior, we found that risk behavior was prevalent during the entire course of the outbreak. In Sierra Leone, concerns were expressed about the response system, such as fears of calling the national hotline and of chlorine spraying (*36*). Ebola-specific information decreased in intensity after the outbreak, which might have resulted in loss of protective behavior and an increase in misconceptions. It can be assumed that repetition of messages is crucial.

In general, information dissemination during the Ebola epidemic was not unidirectional. For instance, radio channels encouraged listeners to call in and share questions. Communication channels should therefore be placed in a broader context, acknowledging the complex interactions among societal, community, and individual features, which in turn affect knowledge and behavior (*37*).

Major strengths of this study are the random sampling method, the large sample size, and the timing of the data collection. Even though the KAP survey instrument was not validated because of the urgency of the setting, KAPs are an established way of gathering data (*15*), and some of the adapted items have been validated in other contexts (*38*). In the surveys, some questions were asked in an open-ended manner, reducing the likelihood of bias because respondents would name only answers they actually knew. The response rate was high, which also reduced the risk of bias. Clear dose-response associations were found, strengthening the hypothesis that exposure to information sources can influence Ebola-specific knowledge and behavior. Further strengths include the possibility to adjust for potential confounders.

Limitations of the study include the fact that the data came from cross-sectional studies and that, therefore, temporality cannot be established. Consequently, directions of the found associations have to be interpreted with caution, as reverse causality cannot be ruled out. Performing mediation analyses with cross-sectional data can seem controversial because there is no way to properly measure if whether the exposure and mediator happen before the outcome. However, because EVD was a new disease in this region of Africa, Ebola-specific knowledge was low at the start of the outbreak (*29*). Furthermore, various information sources were used to disseminate EVD messages at the beginning of the epidemic. It can therefore be assumed that both the exposure and the mediator came before the outcome.

Another limitation is the inherent high correlation between time and district in an infectious disease outbreak, which was addressed by applying multilevel modeling. A further drawback is that, in contrast to KAP 2–4, KAP 1 contained only 9 out of 14 districts. The results were adjusted for region and district, somewhat mitigating this limitation. Furthermore, as previous studies have shown, there is a risk that self-reported behavior might not reflect actual behavior, but in the given outbreak situation, the survey was deemed to be the most feasible method (*39*). Closed-ended questions could have introduced a bias for the risk behavior outcome, owing to the risk of socially desirable answers. To minimize this factor, respondents were encouraged to give honest opinions.

Misclassification of the exposure to information sources could have influenced the results. The number and types of interventions during the EVD epidemic might have made it difficult for respondents to distinguish between information exposures. For instance, lay health workers and community mobilizers might have been viewed as health workers (which was classified as government exposure), overestimating the exposure to that information source (and because of the nondifferential nature of this potential bias, underestimating the results). Last, because exposures were not mutually exclusive during the data collection, a respondent could have been exposed to more than one source of information. To address this factor, for subsequent analyses, mutually exclusive media categories were created.

In conclusion, the results of this study show the importance of information sources in influencing knowledge and behavior—in both positive and negative directions—and could be used for communication strategies in emergency preparedness and disease outbreaks in low-income settings. Our findings underscore the value of risk communication for rapid disease control efforts and, therefore, for global health security.

Technical Appendix Additional information about knowledge, attitude, and practice surveys in Sierra Leone during the Ebola disease outbreak, 2014–2015.
