# Supplementary material for: Risk Communication and Ebola-Specific Knowledge and Behavior during 2014–2015 Outbreak, Sierra Leone
Source: Emerg Infect Dis. 2018 Feb;24(2):336–44. doi: 10.3201/eid2402.171028 (PMC5782897; doi:10.3201/eid2402.171028)
Supplement: Technical Appendix — Additional information about knowledge, attitude, and practice surveys in Sierra Leone during the Ebola disease outbreak, 2014–2015. [file 17-1028-Techapp-s1.pdf]

Article DOI: https://dx.doi.org/10.3201/eid2402.171028

Risk Communication and Ebola-Specific Knowledge and Behavior during the 2014–15 Outbreak, Sierra Leone

Technical Appendix

Technical Appendix Table 1. KAP* sample sizes and response rates, Sierra Leone, 2014–2015

Survey	No. of households	No. of consented respondents	No. of participants approached	Response rate
KAP 1	706	1,413	1,460	97%
KAP 2	1,043	2,087	2,120	98%
KAP 3	1,770	3,540	3,640	97%
KAP 4	1,782	3,564	3,640	98%
Total	5,301	10,604	10,860	98%

*KAP, knowledge, attitude, and practice.

Technical Appendix Table 2. Level of Ebola virus disease outbreak by region, Sierra Leone, 2014–2015

Survey	North	South	West	East
KAP 1*	Before peak	Before peak	Before peak	Peak
KAP 2	Peak	Low after peak	Peak	Low after peak
KAP 3	Low after peak	Low after peak	Low after peak	Low after peak
KAP 4	Low after peak	No transmission	Low after peak	No transmission

*KAP, knowledge, attitude, and practice.

Technical Appendix Table 3. Knowledge and behavior questions in the KAP* surveys, Sierra Leone, 2014–2015

Knowledge questions	Correct answers	Incorrect answers
Open questions		
What causes Ebola?	1. Virus<br>2. Bats/monkeys/chimpanzees	1. God or higher power<br>2. Witchcraft<br>3. Evildoing/sin<br>4. Curse
What happens if someone suspected of Ebola goes to the hospital?	They will take care of him/her (rehydrate, give medicine/food, monitor status)	1. They won’t be able to do anything, they may die there<br>2. They will definitely cure Ebola<br>3. They will find a way to kill the patient<br>Extra KAP 2–4:<br>4. They will be turned away
Closed questions		
Can I prevent myself from getting Ebola by avoiding funeral/burial rituals that require handling the body of someone who died from Ebola?	Yes	No
If a person has Ebola, does he/she have a higher chance of survival if he/she goes immediately to a health facility?	Yes	No
If a person with Ebola goes immediately to a health facility will he/she reduce the chance of spreading it to family or people living with?	Yes	No

Knowledge questions	Correct answers	Incorrect answers
Do you believe that traditional healers can treat Ebola successfully?	No	Yes
Do you believe that spiritual healers can treat Ebola successfully?	No	Yes
Maximum score	KAP 1–4: 8	KAP 1: 12 KAP 2–4: 13
Cutoff	KAP 1–4: 0–6/7–8	KAP 1–4: 0/> = 1

Behavior questions	Correct answers	Incorrect answers
In what ways have you changed your behavior or took actions to avoid being infected with Ebola?	1. I wash my hands with soap and water<br>2. I clean my hands with other disinfectants<br>3. I try to avoid crowded places<br>4. I wear gloves<br>5. I try to avoid physical contact with people I suspect may have Ebola<br><u>Extra in KAP 2–4:</u><br>6. I do not participate in burials that involve handling the dead body<br>7. I use a condom when having sex with an Ebola survivor	1. I was my hands with just water<br>2. I drink Bittercola<br>3. I drink a lot of water/juice<br>4. I drink traditional herbs<br>5. I take antibiotics<br><u>Extra KAP 2–4:</u><br>6. I wash myself with salt and hot water
What would you do if you suspect someone in your family has Ebola?	1. Avoid all physical contact and bodily fluids of that person<br>2. Call the hospital/Ebola phone line	1. Nothing<br>2. Help care for them at home<br>3. Check temperature by touching the body<br>4. Take to the hospital<br><u>Extra KAP 2–4:</u><br>5. Hide them
Maximum correct answers	KAP 1: 7 KAP 2–4: 9	KAP 1: 9 KAP 2–4: 11
Cutoff	KAP 1: 0–2/3–7 KAP2–4: 0–3/4–9	KAP 1–4: 0/> = 1

*KAP, knowledge, attitude, and practice.

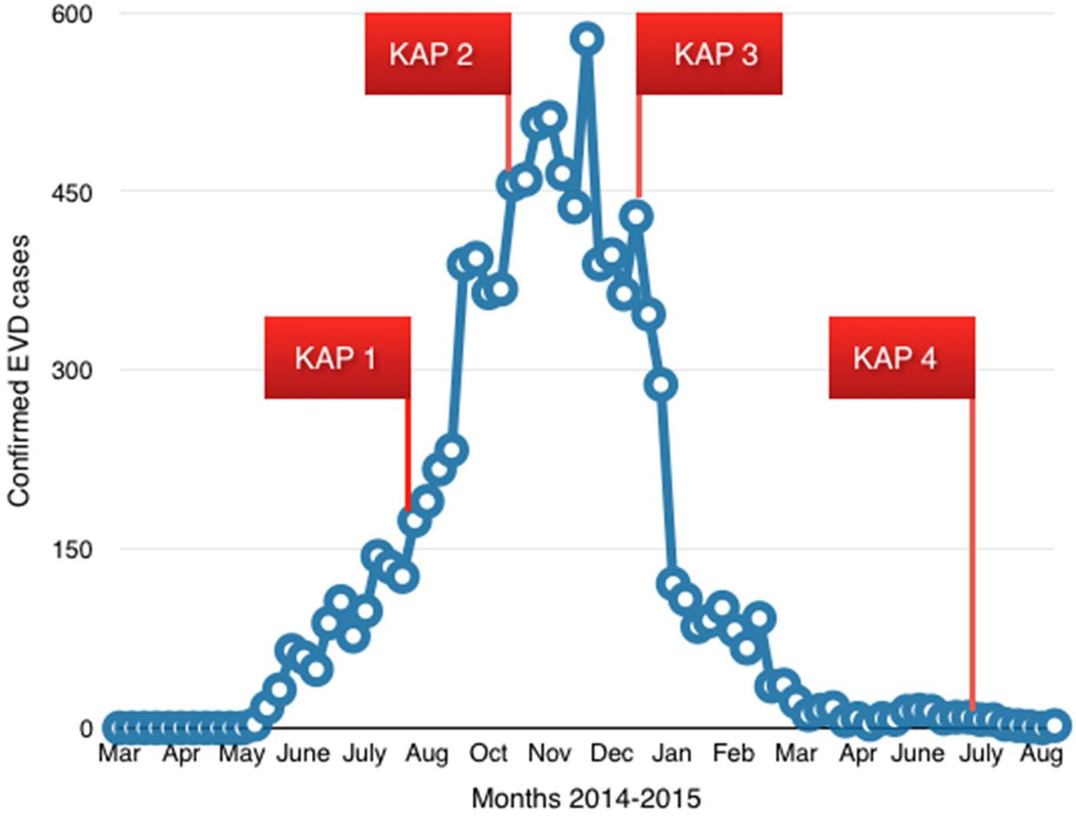

Technical Appendix Figure 1. Overview of time points of knowledge, attitude, and practice (KAP) surveys during the Ebola virus disease (EVD) outbreak in Sierra Leone, 2014–2015.

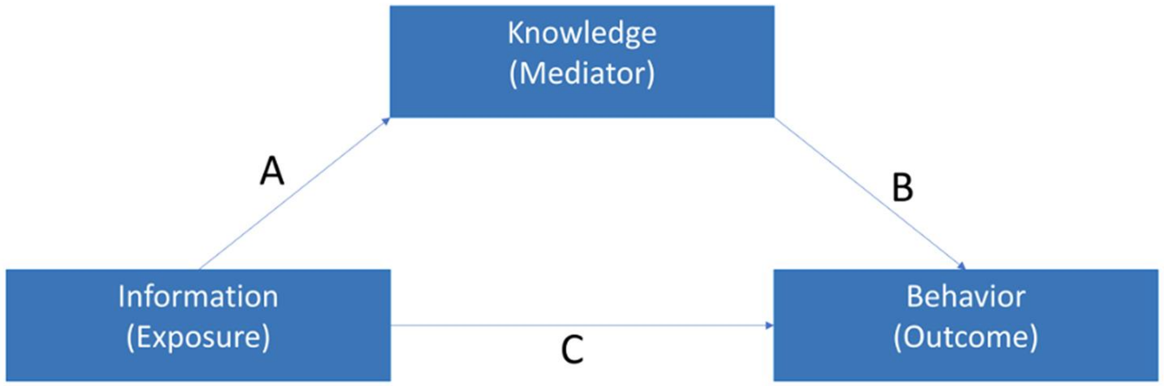

Technical Appendix Figure 2. Mediation analysis among information exposure, Ebola-specific knowledge, and behavior, Sierra Leone, 2014–2015.
